# p53/PD-L1 co-expression predicts poor prognosis in diffuse large B-cell lymphoma

**DOI:** 10.1007/s12672-025-03062-5

**Published:** 2025-07-06

**Authors:** Yaoyao Jing, Ying Yuan, Yong Yu, Haifeng Zhao, Hongliang Yang, Xiaofang Wang

**Affiliations:** 1 Department of Day Ward, Tianjin Medical University Cancer Institute & Hospital, Tianjin, China; 2 Tianjin Medical University Cancer Institute & Hospital, National Clinical Research Center for Cancer, Tianjin, China; 3 Tianjin’s Clinical Research Center for Cancer, Tianjin, China; 4 Key Laboratory of Cancer Prevention and Therapy, Tianjin, China; 5 Department of Hematology, Tianjin Medical University Cancer Institute & Hospital, Tianjin, China; 6 Department of Oncology, Tongren Hospital, Shanghai Jiao Tong University School of Medicine, Shanghai, China

**Keywords:** p53/PD-L1 co-expression, p53, PD-L1, Diffuse large B-cell lymphoma, Prognosis, Rituximab

## Abstract

**Objective:**

To investigate the clinical characteristics and survival of p53 and Programmed death-ligand 1 (PD-L1) co-expression in diffuse large B-cell lymphoma (DLBCL) patients.

**Methods:**

Immunohistochemistry (IHC) was used to detect the expression of p53 and PD-L1 in tumor cells of 176 patients with DLBCL. Clinical data were retrospectively examined along with long-term follow-up. Correlation between the expression of p53 and PD-L1 and the clinicopathological characteristics of the patients was assessed. Impact of Rituximab (R) on prognosis among DLBCL patients with positive expression of p53 and PD-L1 was evaluated.

**Results:**

p53 expression, PD-L1 expression, and p53/PD-L1 co-expression were present in 44.9%, 42.0%, and 25.6% of patients with DLBCL, respectively. No significant differences existed in the clinicopathological characteristics between patients with positive and negative p53 expression and between patients with positive and negative PD-L1 expression. However, more co-expression of p53/PD-L1 was observed in patients with non–germinal center B-cell-like (non-GCB) subtypes (*p* = 0.005). There was a significant positive correlation between p53 and PD-L1 expression (*r* = 0.273, *p* < 0.001). Survival analysis indicated that patients exhibiting p53/PD-L1 co-expression had decreased progression-free survival (PFS) and overall survival (OS ) compared to those demonstrating positive expression of either p53 or PD-L1, and in addition, both negative p53/PD-L1 expression. Rituximab failed to substantially modify the prognosis of patients. p53/PD-L1 co-expression is an independent indicator of adverse prognosis.

**Conclusions:**

Co-expression of p53/PD-L1 in DLBCL patients implies an unfavorable prognostic group, which doesn’t derive benefit from Rituximab.

**Supplementary Information:**

The online version contains supplementary material available at 10.1007/s12672-025-03062-5.

## Introduction

Diffuse large B-cell lymphoma (DLBCL) represents the predominant lymphoma classification globally, comprising 30 to 40% of the total non-Hodgkin lymphomas (NHL) [1, 2]. Despite Rituximab (R) having brought substantial progress in the management of DLBCL, with over half of sufferers attaining enduring remission post first-line chemotherapy, 30 to 40% encounter therapeutic failure due to recurrence or primary drug resistance, primarily amongst high-risk patients [3]. Therefore, exploration of additional biomarkers is crucial in refining management, evaluating high-risk patients, and advancing therapeutic strategies.

In recent years, Programmed death-1 (PD-1) has received widespread attention alongside Programmed death-ligand 1 (PD-L1) pathway. PD-L1 reversibly inhibit the activity and proliferation of T cells by binding to PD-1, thereby protecting normal tissues from immune-mediated injury. However, tumor cells can exploit this pathway to regulate immune response negatively and evade host immune surveillance [4–6]. Previous research have reported that PD-L1 and PD-1 can be identified in primary DLBCL tumor tissues [7–9]. Given that the protein expression of PD-1 plus PD-L1 promotes the development and progression of cancers, inhibitors targeting the immune checkpoint of the PD-1/PD-L1 pathway may offer a potential therapeutic strategy for patients with DLBCL [10, 11].

Tumor protein p53 gene (TP53) is recognized as one of the most frequently mutated genes in cancer [12]. Functioning as a tumor suppressor gene, it takes part in numerous aspects of cellular functions, including DNA repair, cell cycle regulation, apoptosis, and senescence [13, 14].About 20% of DLBCL exhibit TP53 mutations [15]. Studies have shown that the half-life period of mutant p53 protein is longer than that of wild-type p53 protein [16, 17].Consequently, the presence of p53 protein expression identified through immunohistochemistry becomes an indicator for the existence of TP53 mutations.

Studies have shown that PD-L1 can be regulated by p53 via the miR-34 pathway in non-small cell lung cancers [18]. However, in DLBCL, an absence of correlative research on the influence of PD-L1 protein expression and p53 protein expression on the disease remains. The current study delineated the association of PD-L1 and p53 protein expression with clinical features and patient survival in DLBCL.

## Materials and methods

### Patients

This study was a retrospective cohort study encompassed 176 newly diagnosed DLBCL cases at the Tianjin Medical University Cancer Institute & Hospital, subjected to epirubicin plus cyclophosphamide plus vincristine plus prednisone (CHOP ) or CHOP-like chemotherapy (with or without Rituximab) between April 2011 and October 2014. Diagnosis were aligned with the standards defined by the World Health Organization (WHO) 5th edition [19]. Our institution’s pathologists confirmed the histopathological diagnosis for all patients. Patients with a history of malignant tumors, positive EBV or HIV markers were excluded. Clinical characteristics were obtained from patient’s medical records. All patients received at least 4 rounds of standard-dose CHOP or CHOPE (CHOP plus etoposide) regimen with or without Rituximab. The median follow-up period was 55 months (range, 6-214 months). Studies involving human participants were approved and reviewed by the Medical Ethics Committee of Tianjin Medical University Cancer Institute & Hospital.

### Immunohistochemistry

We executed immunohistochemical staining utilizing a two-step immunohistochemistry detection system (PV-9000; Beijing ZSGB-Bio, Beijing, China). Firstly, 4-µm thick paraffin tissue sections were dried at 65℃ for 60 min and subsequently dewaxed and rehydrated via xylene and graded ethanol (95% alcohols, 85% alcohols, 70% alcohols, 5 min each). Antigen extraction was performed in citric acid buffer (with a pH value of 6.0) in pressurized cooker at 220 °C for 2.5 min. Next, slides were immersed in 3% hydrogen peroxide for 20 min to prevent any endogenous peroxidase function. In accordance with instructions issued by the manufacturer, the following antibodies were used: p53 mouse anti-human monoclonal antibody (clone PAb 1801, Abcam, diluted 1:1000) [20, 21] and PD-L1 rabbit anti-human monoclonal antibody (clone 28 − 8, bcam, diluted 1:200) [22, 23]. Incubation was for one night at 4 degrees Celsius. Subsequently, slides were placed in incubation with secondary anti-rabbit (PV-9000; Beijing ZSGB-Bio, Beijing, China) at 37 degrees Celsius for half an hour. Following this, slides were treated with DAB chromogenic solution (Beijing ZSGB-Bio, Beijing, China) and re-dyed with hematoxylin. The latter procedure was followed by dehydration and sealing with neutral gum (Beijing ZSGB-Bio, Beijing, China).

### Staining interpretation

p53 protein is predominantly localized within the nucleus. Expression of the p53 protein was considered confirmed if staining of nuclei was observed among 10% or higher of the lymphoma cells [24–27]. PD-L1 expression typically localizes to tumor cell membranes or cytoplasm [28, 29]. The PD-L1 positive expression was evaluated by calculating the product of the proportions of staining of cells and staining intensity [30]. Positive staining was identified as yellow to brown granules in the cytoplasm or cell membrane. Staining intensity was categorized into four levels: 0: negative; 1: weak; 2: intermediate; 3: strong. Five high power visual fields (× 400 times) containing 100 cells each were randomly read. Stained cell proportion = the quantity of positive cells ÷ the quantity of observed cells×100%, also classified into four grades: 1 = 0–10%; 2 = 10–50%; 3 = 51–75%; 4 = 76–100%. Intensity of staining and proportion of staining multiplication ≥ 3 was counted as PD-L1+. All cases were reviewed independently by two experienced hematopathologists blinded to both the patient’s condition and prognosis.

### Statistical analysis

Overall survival (OS) was defined as the interval between the diagnosis of DLBCL and death from any cause or follow-up (January 2024). Progression-free survival (PFS) was determined by calculating the duration from the date of DLBCL diagnosis to the occurrence of disease progression, relapse, or death from any cause (January 2024). Categorical data comparisons were assessed using chi-square test. Kaplan-Meier method was used for univariate survival analysis and Cox regression model was used for multivariate survival analysis. The difference was considered statistically significant at *p* < 0.05. Statistical analysis was performed using SPSS 27.0 and R software (version 4.2.2).

## Results

### Clinicopathological characteristics

As summarized in Tables 1 and 2, the median age of the cohort was 58 years, with approximately one-third of patients over 60. Over half were male, and 50% had advanced-stage (III/IV) disease. Most patients lacked B symptoms and had low and low-intermediate international prognostic index (IPI) scores. The proportion of germinal center B-cell-like (GCB) and non-GCB subtypes was nearly equal. More than half received Rituximab-based therapy.

**Table 1:** Clinicopathological characteristics of PD-L1 and p53 expressions in DLBCL (*n* = 176)

	p53 expression			PD-L1 expression			p53/PD-L1		
Characteristic	(+), %	(-), %	*P*	(+), %	(-), %	*P*	Co-expression, %	Others, %	*P*
Age (years)									
> 60	31(39.2)	33(34.0)	0.474	29(39.2)	35(34.3)	0.507	19(42.2)	45(34.4)	0.344
≤ 60	48(60.8)	64(66.0)		45(60.8)	67(65.7)		26(57.8)	86(65.6)	
Sex									
Male	46(58.2)	52(53.6)	0.539	42(56.8)	56(54.9)	0.807	26(57.8)	72(55.0)	0.743
Stage									
I–II	38(48.1)	50(51.2)	0.649	41(55.4)	47(46.1)	0.222	25(55.6)	63(48.1)	0.388
III–IV	41(51.9)	47(48.5)		33(44.6)	55(53.9)		20(44.4)	68(51.9)	
B symptoms									
Yes	25(31.6)	24(24.7)	0.309	21(28.4)	28(27.5)	0.892	13(28.9)	36(27.5)	0.856
IPI score									
< 3	46(58.2)	61(62.9)	0.529	44(59.5)	63(61.8)	0.757	24(53.3)	83(63.4)	0.235
≥ 3	33(41.8)	36(37.1)		30(40.5)	39(38.2)		21(46.7)	48(36.6)	
LDH									
Elevated	42(53.2)	53(54.6)	0.845	40(54.1)	55(53.9)	0.986	23(51.1)	72(55.0)	0.655
β2M									
Elevated	25(31.6)	23(23.7)	0.240	18(24.3)	30(29.4)	0.454	15(33.3)	33(25.2)	0.290
COO									
GCB	36(45.6)	58(59.8)	0.060	34(45.9)	60(58.8)	0.091	16(35.6)	78(59.5)	0.005*
Non-GCB	43(54.4)	39(40.2)		40(54.1)	42(41.2)		29(64.4)	53(40.5)	
Extranodal site									
≤ 1	44(55.7)	49(50.5)	0.493	42(56.8)	51(50.0)	0.375	26(57.8)	67(51.1)	0.442
> 1	35(44.3)	48(49.5)		32(43.2)	51(50.0)		19(42.2)	64(48.9)	
Rituximab									
Yes	43(54.4)	60(61.9)	0.320	41(55.4)	62(60.8)	0.475	23(51.1)	80(61.1)	0.242

IPI: international prognostic index, LDH: lactate dehydrogenase, β2M: β2-microglobulin, COO: cell of origin, GCB: germinal center B-cell-like; * *p* < 0.05

### Expression of p53 and PD-L1 in DLBCL

**Fig. 1:**
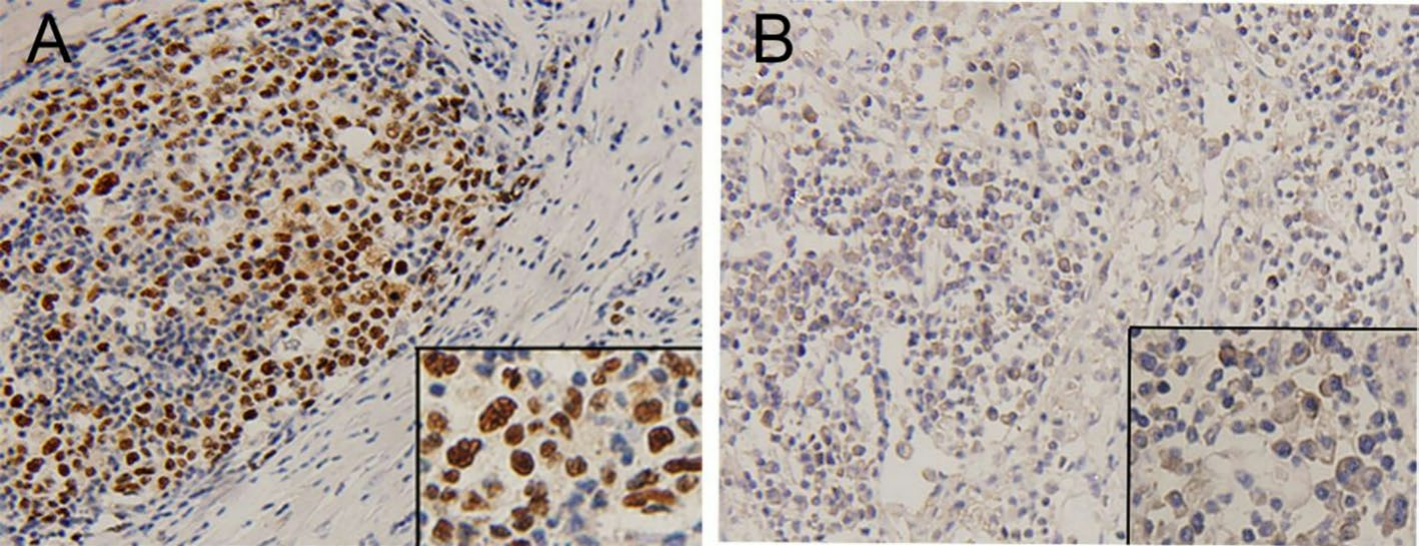
Immunohistochemical staining of p53 and PD-L1 expression in DLBCL tumour tissues. (A) Representative patterns of positive p53 expression (x200 and x400). (B) Representative patterns of positive PD-L1 expression (x200 and x400)

In this study, p53 expression was mainly located in the tumor nucleus (Fig. 1A), whereas PD-L1 protein expression was predominantly located on the membrane and cytoplasm of malignant cells (Fig. 1B). Based on the assessment criteria, the expression rates of p53 and PD-L1 within DLBCL tissues were 44.9% (79/176) and 42.0% (74/176), respectively, yielding a co-expression of 25.6% (45/176), as illustrated in Table 2.

### Clinicopathological analysis of p53 and PD-L1 expression in DLBCL

Table 1 presents the correlation between PD-L1 and p53 protein expression and clinical parameters of DLBCL patients. Findings indicate that while no statistically significant differences were identified, p53 expression within DLBCL tumor cells may potentially be more prevalent in non-GCB subtypes (*p* = 0.06). Nevertheless, no marked disparity in clinical parameters was identified between patients possessing positive and negative PD-L1 expression. We performed further analysis on the clinical relevance of PD-L1 and p53 protein co-expression within DLBCL and found that p53/PD-L1 co-expression was correlation with non-GCB subtypes (*p* = 0.005).

### Prognostic significance of p53 and PD-L1 expression in DLBCL

**Fig. 2:**
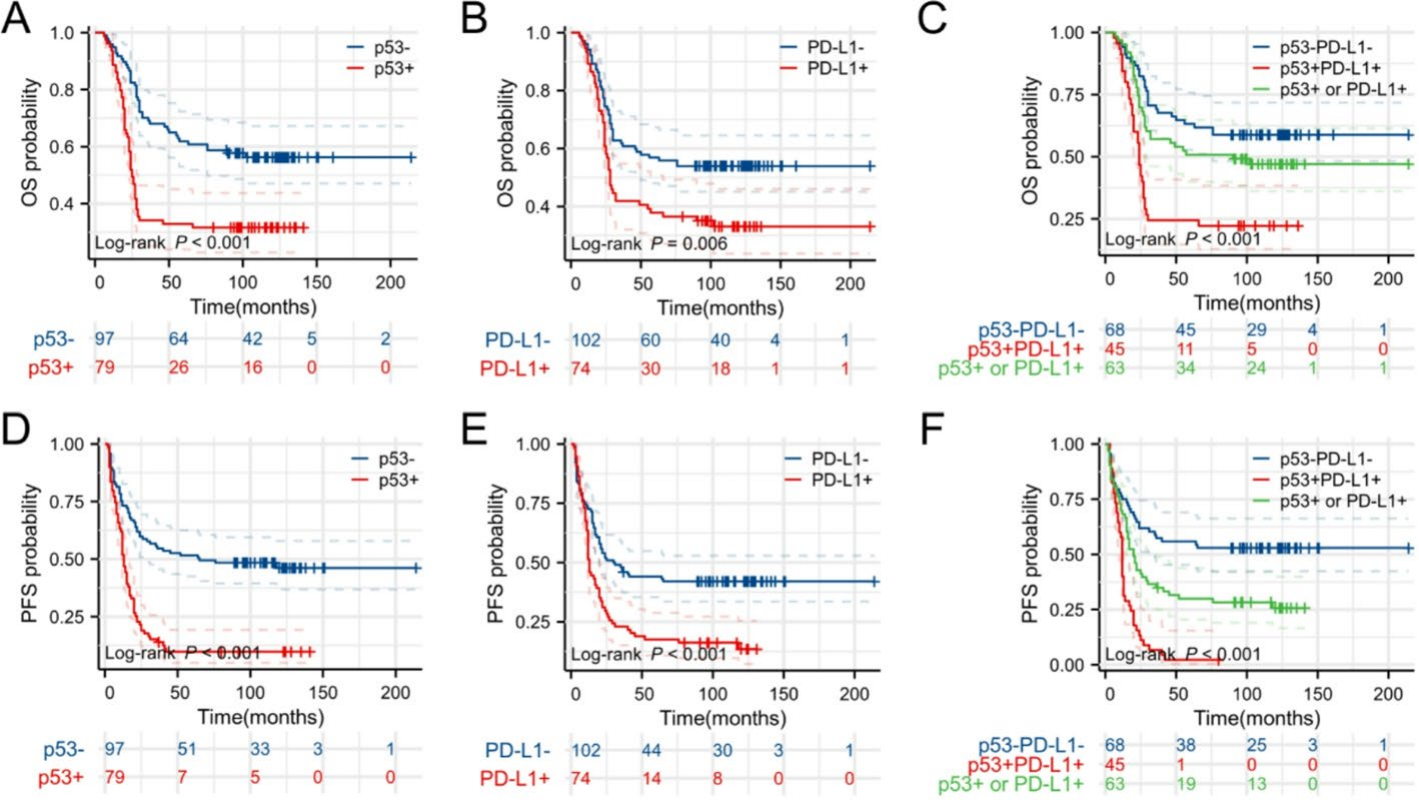
Survival of 176 DLBCL patients according to p53 and PD-L1 expression. (A, D) DLBCL patients of p53 positive expression had a significantly poorer OS and PFS than p53 negative expression. (B, E) PD-L1 positive expression had worse OS and PFS than patients with PD-L1 negative expression. (C, F) Patients with p53/PD-L1 co-expression had a shorter OS and PFS than single positive expression of PD-L1 or p53 and both-negative group

We performed a survival analysis on 176 DLBCL patients. The median follow-up duration for the entire cohort was 55 months (range: 6-214 months). We stratified the entire cohort into three subgroups based on the expression patterns of p53 and PD-L1: p53 expression subgroup, PD-L1 expression subgroup, and p53/PD-L1 co-expression subgroup. The median survival time for patients in these three subgroups was 25 months, 28 months, and 24 months, respectively. As shown in Fig. 2, the subgroup analysis demonstrated that the PFS and OS of DLBCL patients with p53 + were noticeably poorer than those with negativity (Fig. 2A, D). Comparable outcomes were also observed in the PD-L1 subgroup (Fig. 2B, E). In the p53/PD-L1 co-expression subgroup, patients with p53/PD-L1 co-expression exhibited the poorest prognosis, whereas patients with p53/PD-L1 negativity experienced the most favorable outcome (Fig. 2C, F).

### Correlation between p53 and PD-L1 expression

Spearman correlation analysis demonstrated a significant positive correlation between p53 and PD-L1 expression (*r* = 0.273, *p* < 0.001) (Supplementary Table 1).

### Prognostic factors of the entire cohort

Univariate prognostic K-M analysis indicated that patients aged over 60, those scoring IPI 3–5, displaying B symptoms, elevated β2M levels, absence of Rituximab therapy, p53 expression, PD-L1 expression, and p53/PD-L1 co-expression were linked to a poorer OS and PFS. Elevated LDH levels were associated with a diminished OS, yet not PFS (Table 2).

**Fig. 3:**
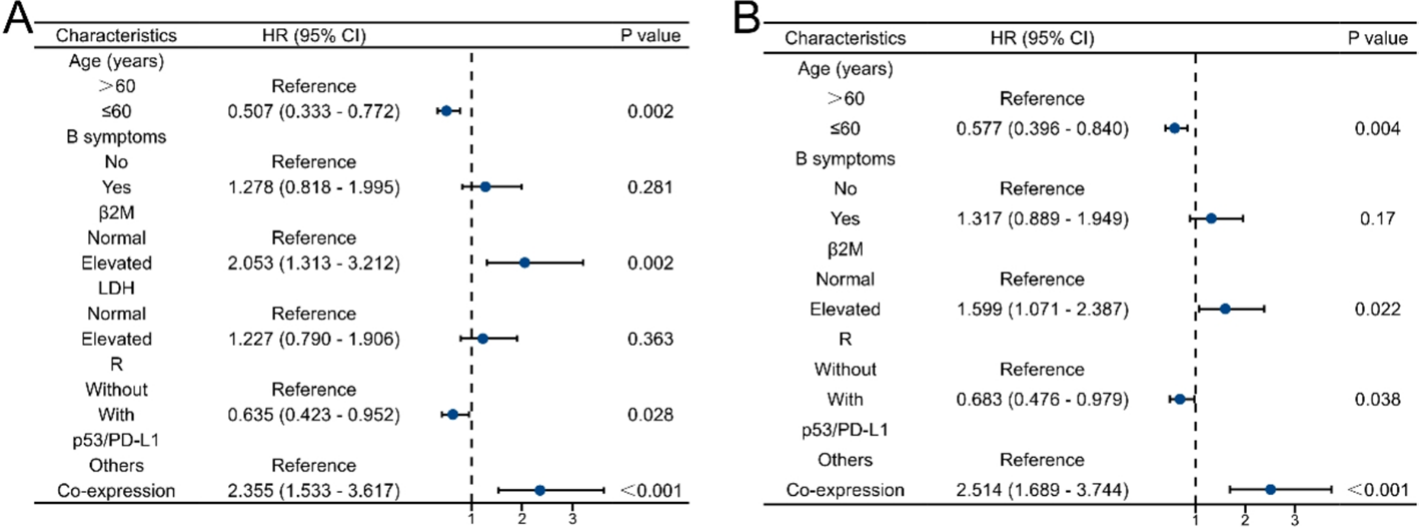
Multivariate analysis of prognostic factors in DLBCL. (A) OS. (B) PFS

Multivariate analysis demonstrated that age greater than 60 years, the presence of B symptoms, elevated β2M level levels, absence of Rituximab therapy, and p53/PD-L1 co-expression were independent indicators of inferior OS and PFS in DLBCL patients (Fig. 3).

It should be noted that although there were significant statistical variances observed for IPI scores, p53 expression, and PD-L1 expression in the univariate analysis, these indicators were omitted from the multivariate analysis of PFS and OS to address the potential collinearity bias as they were highly correlated with the assessed indicators.

**Table 2:** Univariate analysis of prognostic factors in DLBCL

Characteristic	*n* (%)	*P*	
		OS	PFS
Age (years)		<0.001*	<0.001*
> 60	64 (36.4)		
≤ 60	112 (63.6)		
Sex		0.113	0.522
Male	98 (55.7)		
Stage		0.095	0.112
I−II	88 (50.0)		
III−IV	88 (50.0)		
B symptoms		0.027*	0.043*
Yes	49 (27.8)		
IPI score		<0.001*	<0.001*
< 3	107 (60.8)		
≥ 3	69 (39.2)		
LDH		0.016*	0.054
Elevated	95 (54.0)		
β2M		<0.001*	<0.001*
Elevated	48 (27.3)		
COO		0.187	0.402
GCB	94 (53.4)		
Non-GCB	82 (46.6)		
Extranodal site		0.066	0.226
≤ 1	93 (52.8)		
> 1	83 (47.2)		
Rituximab		0.050*	0.045*
Yes	103 (58.5)		
p53 expression		<0.001*	<0.001*
Positive	79 (44.9)		
PD-L1 expression		0.006*	<0.001*
Positive	74 (42.0)		
p53/PD-L1		<0.001*	<0.001*
Co-expression	45 (25.6)		
Others	131 (74.4)		

IPI: international prognostic index, LDH: lactate dehydrogenase, β2M: β2-microglobulin, COO: cell of origin, GCB: germinal center B-cell-like; * *p* < 0.05

### Prognostic significance of rituximab in patients with the protein expression of p53 and PD-L1

**Fig. 4:**
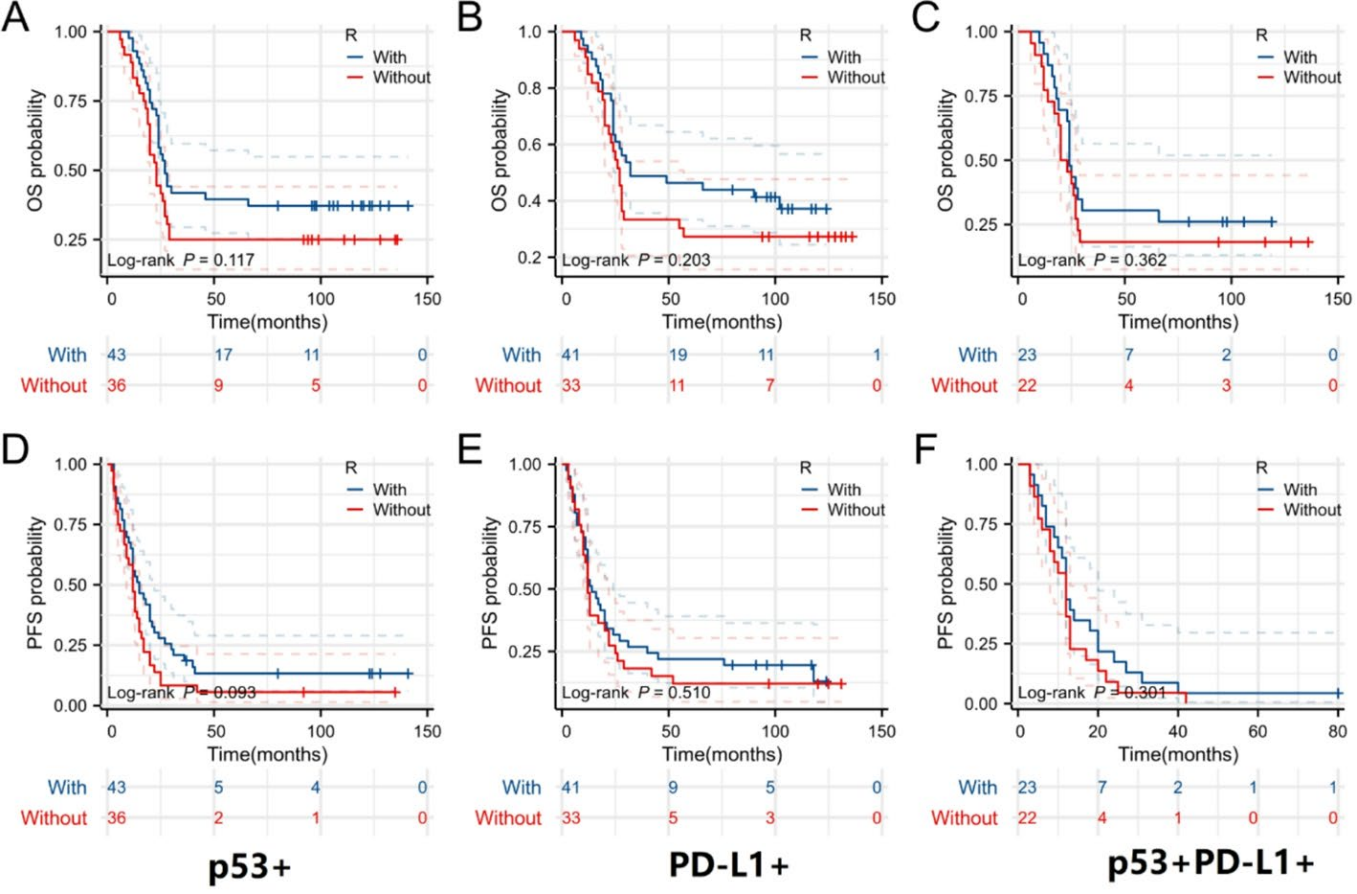
Prognostic significance of Rituximab in patients with the expression of p53 and PD-L1. For patients with p53 expression (A, D), PD-L1 expression (B, E), and p53/PD-L1 co-expression (C, F), treated with Rituximab did not have a survival benefit

As indicated in Table 1, the percentages of patients prescribed R-CHOP or R-CHOP-like therapies within the three subgroups of p53 expression, PD-L1 expression, and p53/PD-L1 co-expression were 54.4% (43/79), 55.4% (41/74), and 51.1% (23/45), respectively. There was no noteworthy disparity in the use of Rituximab across the three subgroups. Furthermore, analysis revealed that patients in these three subgroups were unable to gain benefit from Rituximab (Fig. 4A-F).

## Discussions

p53 and PD-L1 exhibit high expression in multiple malignancies including DLBCL, with a noted correlation of their expression in lung cancer [31, 32]. Nonetheless, the predictive value of p53/PD-L1 protein co-expression in DLBCL has not been elucidated. This investigation intends to ascertain the expression of p53 and PD-L1 protein in DLBCL, along with the prognostic relevance of p53 and PD-L1 protein expression in DLBCL patients.

Genomic data acquired from over 20,000 patients have confirmed that TP53 is the most predominant mutated gene in all human malignancies [12, 33–35], such as lymphoma [15], hepatocellular carcinoma [36, 37], colorectal cancer [38], and mucosal melanoma [39]. In recent years, immunotherapy targeted at PD-1/PD-L1 pathway has emerged as an efficacious strategy for lots of advanced malignancies [4, 6, 40, 41]. Growing evidence highlights a correlation between TP53 mutations and PD-L1 expression. TP53 mutations regulates immune evasion through controlling PD-L1 expression in neoplastic cells [42]. Based on the genomic, transcriptomic, proteomic, and clinical cancer database in non-small cell lung cancer and lung adenocarcinoma, individuals with TP53 mutations or TP53 mutation-associated epithelial-mesenchymal transition (EMT) phenotype exhibit elevated PD-L1 mRNA expression and diminished miR-34/miR-200 [31, 43–46]. Both miR-34 and miR-200 can inhibit PD-L1 by specifically binding to the PD-L1 13 ′-UTR [47]. TP53 mutations mitigate miR-34/miR-200 expression in EMT, thereby propelling high levels of PD-L1 expression in non-small cell lung cancer. Nevertheless, elevated PD-L1 has demonstrated considerable benefits for PD-1/PD-L1 blockade, and p53 may also serve as a potential steer in selecting immunotherapy [31, 43]. Moreover, p53 could trigger a series of responses that include the constitutive activation of NF-kB and the damage of plasma cell terminal differentiation [48]. Although current studies often define abnormal p53 expression patterns as either diffuse strong positivity in nearly all tumor cells (overexpression pattern) or a complete absence (null pattern) to indirectly reflect underlying TP53 mutation status, several studies have indicated that even lower immunohistochemical positivity thresholds (such as > 10% or 20%) for p53 expression can still significantly predict survival outcomes in patients with DLBCL [26, 49]. Our findings are consistent with these previous studies. Despite the escalating correlation between TP53 mutation and PD-L1 expression, the clinical significance of co-expression of p53 protein and PD-L1 in DLBCL remains largely unexplored. Therefore, this investigation elected patients with DLBCL as the research subjects to probe the potential nexus between p53/PD-L1 co-expression and the clinical characteristics and prognosis of DLBCL. Subgroup analysis revealed that the p53/PD-L1 co-expression cohort displayed the poorest prognosis, which was an independent poor prognostic factor. Furthermore, from the pathological features, p53/PD-L1 co-expression was more frequent in non-GCB subtypes. Put succinctly, our study elucidates that p53/PD-L1 co-expression constitutes a novel subtype associated with unfavorable prognosis in DLBCL patients. Future steps of our study are to expand case to corroborate or refute these conjectures and potential mechanisms.

PD-1 is predominantly expressed on B cells, activated T cells, and natural killer (NK) cells [50, 51], whilst the PD-1 ligand PD-L1 is displayed in tumor cells and immune cells [4, 52]. PD-L1 is absent from normal epithelial tissue but aberrantly expressed in numerous solid cancer cells and is correlated with unfavorable prognosis [53–56]. In many solid neoplasms, it is the expression of PD-L1 rather than PD-1 which correlates with a patient’s response to PD-1 inhibitors and patient prognosis [57–60]. However, in lymphoid malignancies, the scenario is more intricate. Expression of PD-L1 in tumor cells has been reported in some types of lymphomas such as extranodal NK/T cell lymphoma (ENKTL) [61–63], but not in certain types such as Burkitt lymphoma, nodular lymphocyte-predominant Hodgkin lymphoma, and HHV8-associated Kaposi’s sarcoma [64]. For DLBCL, the expression level of PD-L1 in tumor cells has also been reported variably. Some reports indicated that elevated levels of PD-L1 expression can be observed in DLBCL tumor cells compared with FL tumor cells [65], while other studies suggest that PD-L1 expression levels are typically low in B-cell NHL tumor cells, and can only be observed in some non-GCB subtypes of DLBCL [66–69]. Some potential mechanisms may contribute to PD-L1 overexpression in DLBCL, such as genetic alterations of 9p24.1 or activation of the JAK/STAT pathway [70, 71]. These mechanisms may act independently or in concert and warrant further investigation in the context of DLBCL immune evasion. Moreover, the prognostic significance of PD-L1 in DLBCL remains contentious. Some studies find no correlation between the expression of PD-L1 in tumor cells and OS in DLBCL patients [72, 73], while others suggest worse OS for PD-L1 + DLBCL patient**s** [74].The present study employed DLBCL as the subject and verified the negative influence of tumor cell PD-L1 expression on patient prognosis (PFS and OS). This furnishes robust evidence for the forthcoming management of DLBCL patients.

Since the debut of the Rituximab epoch, the prognosis for DLBCL patients has appreciably ameliorated. However, the function of Rituximab in DLBCL patients possessing p53 or PD-L1 expression remains unclear. Our research revealed that those patients harboring p53/PD-L1 co-expression possess an exceedingly poor prognosis, and this poor prognosis is not mitigated by Rituximab. Hence, it is imperative to initiate prospective studies of novel regimens promptly.

We infer that immune checkpoint inhibitors of PD-L1 will constitute a novel and efficacious therapeutic strategy for aggressive DLBCL, and anticipate favourable outcomes similar to those observed in the management of certain solid malignancies [40, 41, 75–77]. The integration of anti-p53 antibody-based targeted therapy with PD-L1 immune checkpoint inhibitors will inaugurate a novel chapter in the therapeutic paradigm for patients afflicted with DLBCL.

This study has several limitations that warrant consideration. Firstly, as a retrospective, single-center study with a relatively limited sample size, our findings require validation by larger, prospective, multicenter studies to confirm their clinical relevance and generalizability. Secondly, the definition of p53 positivity in this study was based on immunohistochemical staining (> 10%), which differs from current recommendations that define aberrant p53 expression as either a “null pattern” (complete absence of staining) or an “overexpression pattern” (diffuse strong positivity in nearly all tumor cells). Therefore, our criteria may not accurately represent the true underlying TP53 mutation status, and caution should be exercised when interpreting the prognostic significance of p53 expression in this context. Finally, our analysis did not explore the detailed molecular mechanisms underlying the observed association between p53 and PD-L1 expression; additional studies are needed to elucidate these mechanisms further.

## Conclusions

In summary, our research affirms that p53/PD-L1 co-expression in patients with DLBCL displays a significant adverse prognostic influence, and that the clinical outcome of this cohort cannot be necessarily improved by incorporating Rituximab into chemotherapy treatment. These findings imply that p53/PD-L1 co-expression may serve as a potentially predictive biomarker for further prognosis stratification of patients within the high-risk group of DLBCL. Patients afflicted with p53/PD-L1 co-expression are prevalent in non-GCB subgroups and exhibit an extremely poor prognosis, thus precise and early identification of this subtype in DLBCL is paramount.

## Electronic supplementary material

Below is the link to the electronic supplementary material.

Supplementary Material 1

## Data Availability

The datasets used and/or analysed during the current study are available from the corresponding author on reasonable request.

## Abbreviations

DLBCL: Diffuse large B-cell lymphoma
IHC: Immunohistochemistry
R: Rituximab
NHL: Non-Hodgkin lymphomas
PD-1: Programmed death-1
PD-L1: Programmed death-Ligand 1
OS: Overall survival
PFS: Progression-free survival
GCB: Germinal center B-cell-like
IPI: International prognostic index
LDH: Lactate dehydrogenase
β2M: β2-microglobulin
COO: Cell of origin
