# Supplementary material for: p53/PD-L1 co-expression predicts poor prognosis in diffuse large B-cell lymphoma
Source: Discov Oncol. 2025 Jul 6;16:1270. doi: 10.1007/s12672-025-03062-5 (PMC12229974; doi:10.1007/s12672-025-03062-5)
Supplement: Supplementary file 1 — Supplementary Material 1 [file 12672_2025_3062_MOESM1_ESM.docx]

**Supplementary Table 1** Univariate analysis of prognostic factors in DLBCL.

|  | **p53 expression** | | *r* | *p* |
| --- | --- | --- | --- | --- |
|  | Positive (n) | Negative (n) |  |  |
| **PD-L1 expression** |  |  |  |  |
| Positive (n) | 45 | 29 | 0.273 | ＜0.001 |
| Negative (n) | 34 | 68 |  |  |
